# Molecular Motors in Myelination and Their Misregulation in Disease

**DOI:** 10.1007/s12035-024-04576-9

**Published:** 2024-10-31

**Authors:** Daniel José Barbosa, Cátia Carvalho, Inês Costa, Renata Silva

**Affiliations:** 1 i3S - Instituto de Investigação e Inovação em Saúde, Universidade do Porto, 4200-135 Porto, Portugal; 2 Associate Laboratory i4HB - Institute for Health and Bioeconomy, University Institute of Health Sciences - CESPU, 4585-116 Gandra, Portugal; 3 UCIBIO - Applied Molecular Biosciences Unit, Translational Toxicology Research Laboratory, University Institute of Health Sciences (1H-TOXRUN, IUCS-CESPU), 4585-116 Gandra, Portugal; 4 ICBAS - Instituto de Ciências Biomédicas Abel Salazar, Universidade do Porto, 4050-313 Porto, Portugal; 5 Associate Laboratory i4HB - Institute for Health and Bioeconomy, Faculty of Pharmacy, University of Porto, 4050-313 Porto, Portugal; 6 UCIBIO - Applied Molecular Biosciences Unit, Laboratory of Toxicology, Department of Biological Sciences, Faculty of Pharmacy, University of Porto, 4050-313 Porto, Portugal

**Keywords:** Myosins, Kinesins, Dynein, Myelination, Demyelinating diseases, Multiple sclerosis

## Abstract

Molecular motors are cellular components involved in the intracellular transport of organelles and materials to ensure cell homeostasis. This is particularly relevant in neurons, where the synaptic components synthesized in the soma need to travel over long distances to their destination. They can walk on microtubules (kinesins and dyneins) or actin filaments (myosins), the major components of cell cytoskeleton. While kinesins mostly perform the anterograde transport of intracellular components toward the plus ends of microtubules located distally in cell processes, cytoplasmic dyneins allow the retrograde flux of intracellular cargo toward the minus ends of microtubules located at the cell soma. Axon myelination represents a major aspect of neuronal maturation and is essential for neuronal function, as it speeds up the transmission of electrical signals. Increasing evidence supports a role for molecular motors in the homeostatic control of myelination. This role includes the trafficking of myelin components along the processes of myelinating cells and local regulation of pathways that ensure axon wrapping. Dysfunctional control of the intracellular transport machinery has therefore been linked to several brain pathologies, including demyelinating diseases. These disorders include a broad spectrum of conditions characterized by pathological demyelination of axons within the nervous system, ultimately leading to axonal degeneration and neuronal death, with multiple sclerosis representing the most prevalent and studied condition. This review highlights the involvement of molecular motors in the homeostatic control of myelination. It also discusses studies that have yielded insights into the dysfunctional activity of molecular motors in the pathophysiology of multiple sclerosis.

## Introduction

Molecular motors are essential for a wide range of functions in dividing and non-dividing cells. In neurons and glial cells, the vesicular transport of organelles and other intracellular cargo along their processes is perhaps their most explored and characterized role [1]. By walking on microtubule tracks, multiple kinesins ensure the anterograde flux of cargo towards synaptic termini, whereas cytoplasmic dyneins (essentially cytoplasmic dynein 1) retrogradely transport intracellular components toward the neuronal soma [2–5]. Alternatively, myosin motors, by walking on actin filaments, also mediate the delivery of intracellular materials to specific locations [6]. Since axonal trafficking is crucial to timely ensure the proper spatial distribution of intracellular components, it is of utmost importance for proper neuronal function. In fact, defects in axonal transport machinery and dynamics have been increasingly associated with several unrelated neurodegenerative and neurological conditions [5]. This suggests that molecular motors are key participants in the homeostatic control of axonal trafficking to secure proper neuronal development, function, and survival.

Axon myelination represents a key event during neuronal development and maturation [7–9]. Molecular motors are important for the delivery of myelin components for myelinating sites [10, 11]. Moreover, they also play a local role in regulating signaling pathways and molecules in those sites [12]. Additionally, molecular motors also support the remodeling of cellular cytoskeleton required for proper axonal wrapping by myelinating cells [13].

Demyelinating diseases represent a large group of pathologies generally characterized by defects in myelin sheaths that surround neuronal axons. They can affect neurons in both central and peripheral nervous systems (CNS and PNS, respectively), resulting in a wide spectrum of clinical manifestations, with variable age of onset, progression, and recovery [14–18]. Among the different demyelinating diseases, multiple sclerosis (MS) constitutes the most prevalent condition. Given the role of molecular motors in neuronal homeostasis and function, increasing evidence has linked perturbations in their activity to MS [19].

In this review, were report the involvement of molecular motors in the homeostatic control of myelination and their involvement in demyelinating diseases, particularly in MS. Thus, we bring up the available data implicating molecular motors in the pathogenesis of this condition.

## Molecular Motors: Generalities

**Fig. 1:**
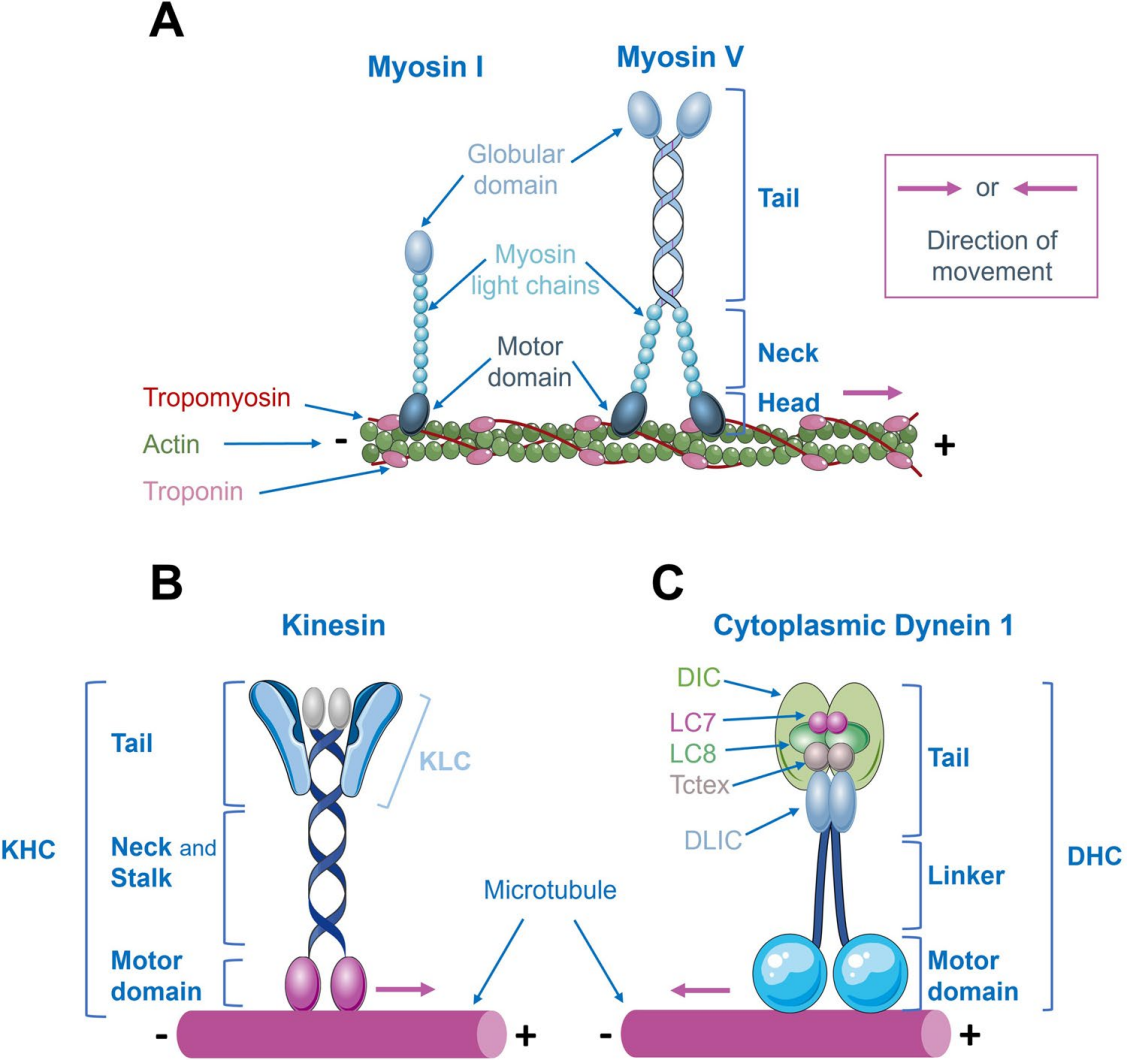
Schematic diagrams of molecular motors. **A** Structure of myosin I and myosin V families. All myosin motors, except myosin VI, move toward the plus ends of actin filaments. The myosin I family of motors functions as a monomer, while conventional myosins, including myosin V, function as dimers. They generally consist of a motor domain and a tail that binds to membranes (myosin I) or organelles (myosin V). **B** Structure of kinesins. They transport cargo along microtubules toward their plus ends (except kinesin-14, which moves toward the minus ends, and kinesin-13 motors that do not move directionally on microtubules). Kinesins are composed of a dimer of Kinesin Heavy Chains (KHC), with motor domains at their N-termini. The neck linkers connect the motor domains to the stalk, and the tail region is involved in binding to Kinesin Light Chains (KLC) and cargo. **C** Structure of cytoplasmic dynein 1, the main microtubule minus-end-directed motor. The complex is built on a dimer of Dynein Heavy Chains (DHC). Dimers of Dynein Intermediate Chains (DIC) and Dynein Light Intermediate Chains (DLIC) bind to DHC. Dimers of the three Dynein Light Chain (DLC) families (Roadblock (Robl/LC7), LC8, and Tctex) bind to the DIC N-terminus

Cells have extraordinarily intricate and dense cytoplasmic regions where the temporal and spatial control of organelle distribution is governed by molecular motors, ensuring proper cell organization and function. Molecular motor complexes associate with polarized cytoskeleton filaments, such as actin and microtubules, and move steadily along them, powered by repeated cycles of adenosine 5’-triphosphate (ATP) hydrolysis. These motor complexes differ in the cargo they transport, the type of filament they bind to, and the direction of movement. Three major classes of motor proteins have been described as cargo transporters: kinesins, dyneins, and myosins [20]. Figure 1 shows schematic diagrams of molecular motors, and Table 1 summarizes their main characteristics and functions. For a detailed exploration of molecular motors, see also [21–29]. Whereas both dyneins and kinesins bind and move along microtubules, myosin motors bind to and move along actin filaments [1].

**Table 1:** Summary information on myosins, kinesins, and dyneins

	Molecular motor superfamily		
	Myosins	Kinesins	Cytoplasmic dyneins
Number	18 classes	40 types	2 types
Cytoskeletal filament	Actin	Microtubules	Microtubules
Directionality	Toward the plus ends^1^	Toward the plus ends^2^	Toward the minus ends
Structural characteristics	- Monomeric (myosin I) - Dimeric complex (conventional myosins) - Composed by a motor domain (hydrolyzes ATP), a neck region (regulates motor activity), and a tail domain (responsible for dimerization, cargo binding, and targeting)	- Dimeric complex of 2 KHC and 2 KLC - KHC—MT-binding ATPase motor domain at its N*-*terminus, neck linkers (connect the motor domains to the stalk, and control the movement and coordination of motor domains), and stalk domain and tail region (responsible for KHC dimerization, complex regulation, and KLC and cargo binding) - KLC—regulates cargo binding	- Dimeric complex of 2 DHCs, 2 DICs, 2 DLICs, and 6 DLCs (dimers of Robl/LC7, LC8, and Tctex) - DHC—has a C-terminal motor domain that hydrolyzes ATP and binds MT, and a stalk and tail region that allow complex dimerization and binding to other subunits and cargoes - DIC, DLIC, DLCs—responsible for cargo binding, and complex regulation and stability
Functions	- Muscle contractility - Membrane trafficking - Signal transduction - Intracellular transport	- Cilia assembly - Intracellular trafficking - Organelle positioning - Microtubule sliding - Regulation of microtubule dynamics	- Cilia transport ^3^ - Intracellular trafficking - Organelle positioning - KT-MT interactions - SAC inactivation - Spindle positioning and orientation

*ATP*, adenosine-5’-triphosphate; *DHC*, Dynein Heavy Chain; *DIC*, Dynein Intermediate Chain; *DLC*, Dynein Light Chain; *DLIC*, Dynein Light Intermediate Chain; *KHC*, Kinesin Heavy Chain; *KLC*, Kinesin Light Chain; *KT*, Kinetochores; *LC8*, Dynein Light Chain 8; *MT*, Microtubules; *Robl/LC7*, Dynein Light Chain Roadblock/7; *SAC*, Spindle assembly checkpoint; *Tctex*, Dynein Light Chain Tctex

^1^Except for myosin VI

^2^Except for kinesin-14 that moves towards the minus-ends, and kinesin-13 motors, which do not move directionally on microtubules

^3^Only cytoplasmic dynein 2

Among these three families of molecular motors, myosin was the first to be identified. In 1864, Kühne described the first myosin (M2, currently termed as conventional myosin or myosin II) [29]. Nowadays, the myosin superfamily includes 18 classes of motors encoded by about 40 myosin genes in the human genome [30]. This classification is primarily based on comparisons and phylogenetic analysis of the conserved motor domain. Structurally, most myosins form a dimer and consist of a motor domain, a neck region, and a tail region. Myosin motor domain, also known as head, binds to and hydrolyses ATP to power movement. Myosin tails have diversified during evolution to permit the proteins to dimerize with other subunits and to interact with different cargoes [29]. All myosin motors, except myosin VI [31], move toward the plus ends of actin filaments. Some myosin motors work as a monomer and are classified in the myosin I family of motors (they have only one head), while conventional myosins were subsequently grouped in the myosin II family (they have two heads) (Fig. 1). Since then, many other myosin types have been discovered. Several one-head and two-head varieties related to myosin I and myosin II families have been described. Nowadays, the nomenclature is based on the order of discovery (myosin III through at least myosin XVIII). Although some of these myosins were found in plants (myosins VIII and XI), others are only found in vertebrates (myosin IX). Interestingly, eucaryotes have most of the described myosins, suggesting that myosins emerged early in eukaryotic evolution [32].

Despite their well-known role in muscle contractility, myosin motors are also important for cell movement, cytokinesis, membrane trafficking, and signal transduction [29]. Particularly, myosin V is involved in intracellular transport, especially in the movement of organelles, such as mitochondria, along actin filaments [33] (Table 1). Myosin VII is also mainly involved in cargo distribution within the actin-rich stereocilia of inner ear hair cells, responsible for hearing [34].

Later on, in 1985, Vale and colleagues identified a new motor complex, kinesin, now known as kinesin-1, in protein extracts from giant axon of the squid [35]. For several years, kinesin-1 stood as the sole known motor capable of transporting cargo along microtubules toward their plus-ends (Fig. 1; Table 1) [28]. Nowadays, the kinesin superfamily is a large and diverse group of motor proteins, with over 40 known types of kinesins identified in humans [36]. These motors have been categorized into 14 different families, based on their protein structures, functions, and evolutionary relationships, although some of them remain ungrouped or are considered orphan kinesins [36, 37]. These motor proteins are composed of a dimer of Kinesin Heavy Chains (KHC), which have motor domains at their N-termini. The neck linkers connect the motor domains to the stalk and control the movement and coordination of the motor domains. The stalk domain facilitates dimerization and forms the elongated structure of the protein, while the tail region, involved in cargo binding, is where a dimer of Kinesin Light Chains (KLC) binds (Fig. 1). The globular motor domain of KHC hydrolyses ATP, allowing the movement of the motor toward the plus ends of microtubules. On the other hand, the kinesin-14 represents an unconventional kinesin that shows minus-end directed movement along microtubule tracks. By contrast, kinesin-13 motors do not move directionally on microtubules, but are important microtubule regulators as they induce microtubule disassembly [38] (Fig. 1; Table 1).

**Fig. 2:**
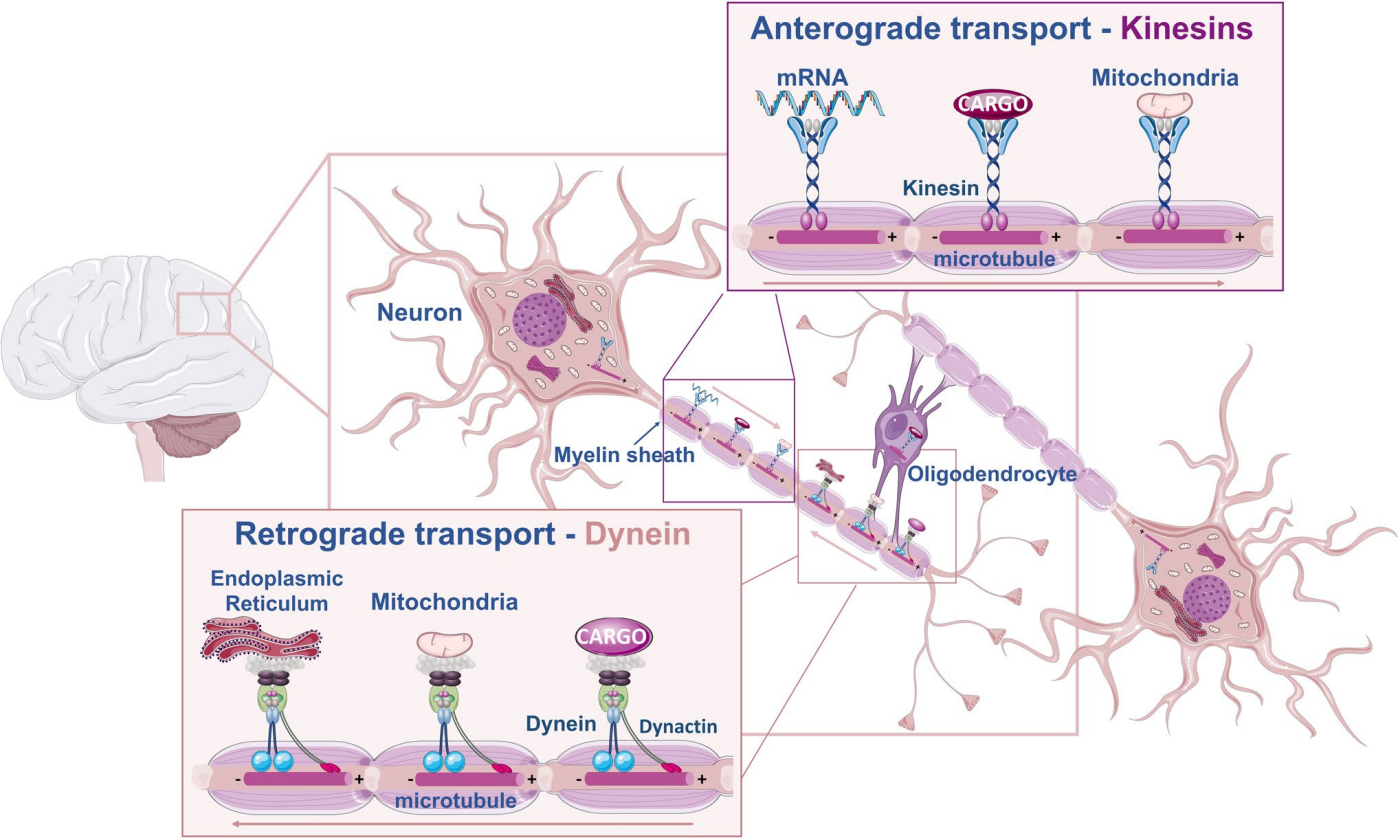
Molecular motors are essential components in brain cells to support the spatiotemporal distribution and localization of organelles and other intracellular cargoes. Both kinesins and dynein (cytoplasmic dynein 1) use microtubule tracks to transport intracellular organelles and materials along neuronal projections. Most kinesins show plus-end directed movement, whereas cytoplasmic dyneins move cargoes toward the microtubules minus ends. In neurons, the transport powered by molecular motors is particularly important for the distribution of organelles and materials (mRNAs) that are synthetized in the soma, and for the recycling and elimination of damaged organelles and other intracellular components that need to be traveled back to the soma. Myelinating cells, which include oligodendrocytes and Schwann cells, are also dependent on molecular motors to ensure the coordinated transport of myelin components to myelination sites

While motor domains show high sequence homology among different kinesins [28, 36], both the stalk domain and tail region show more variability. These two regions are known to recognize and bind to cargoes for their transport. Despite their functions in vesicle transport and organelle positioning, which is crucial for the spatiotemporal distribution of cargo in neurons and glial cells (Fig. 2), and for successful mitotic and meiotic divisions, kinesins are also involved in microtubule sliding and regulate microtubule dynamics. Additionally, kinesin-2 has been implicated in the transport of cilia-assembling precursors that are crucial for the motility of cilia and flagella (Table 1) [39]. Mutations in kinesins and associated proteins have already been associated with neurodegenerative diseases [40].

Right after the discovery of kinesin-1, work from the Vallee’s lab identified another type of molecular motor, initially described as MAP1C, with microtubule-activated ATPase activity capable of translocating microtubules in vitro [41]. Nowadays, this motor is known as cytoplasmic dynein 1. Only two dynein motors are involved in cargo transport in eukaryotic cells: cytoplasmic dynein 1 and cytoplasmic dynein 2 (Table 1) [42]. Despite cytoplasmic dyneins, analogous motors with essential roles in the axonema (cilia and flagella) are classified as axonemal dyneins.

Cytoplasmic dynein 1, the most important and abundant dynein motor (hereafter called dynein), is a 1.4 megadalton complex of six different chains, all organized in dimers [43]. This complex is built on a dimer of Dynein Heavy Chain (DHC), which is encoded by a single human gene (*DYNC1H1*). Dimers of other dynein subunits, including Dynein Intermediate Chain (DIC) and Dynein Light Intermediate Chain (DLIC), bind to DHC. Additionally, dimers of the three families of Dynein Light Chain (DLC) (Roadblock (Robl), LC8, and Tctex) bind to the DIC N-terminus (Fig. 1; Table 1).

Dynein is the main motor responsible for the minus-end-directed movement of cargoes along microtubules (kinesin-14 also powers minus-end directed movement of cargo on microtubule tracks, although with a minor role) (Fig. 1 and Fig. 2). Dynein function is regulated by several cofactors, including dynactin, which is required for almost all dynein functions. Dynactin is a 1.0 megadalton complex that shows microtubule-binding activity through its largest subunit p150^Glued^ [44]. Recent work has shown that cargo-specific adaptor proteins form a three-way complex with dynein and dynactin, allowing highly processive motility in vitro [45–47]. Currently, approximately 25 known or putative activating cargo adaptors are described, 16 of which have been shown to bind DLIC with biological significance [23, 43, 48–50].

Dynein is responsible for the minus-end directed transport of several organelles, such as endosomes, lysosomes, mitochondria, phagosomes, and lipid droplets in dividing and non-dividing cells. Some studies have shown that the disruption of dynein blocks transport between organelles [51] and impairs neuronal retrograde transport [52]. Other dynein functions include the generation of pulling forces on microtubule network that contributes to the positioning of several organelles. In addition, in dividing cells, dynein is responsible for focusing the microtubules minus-ends at the spindle poles, for establishing the interaction between kinetochores and microtubules, for the inactivation of the spindle assembly checkpoint, and for spindle positioning and orientation (Table 1) [53].

## Molecular Motors in Myelination

Myelination of axons of CNS neurons is essential to facilitate the propagation of electrical signals and to support axonal metabolism [54]. This process requires formation of an appropriate number of oligodendrocyte progenitor cells, and their migration from their origin to their target axons, the axonal wrapping by their extended membranes, and the active transport of myelin membrane components [7, 55]. On the other hand, proteins that are required for building the myelin sheaths are synthesized either in the soma of myelinating cells or in their branches [56]. Thus, molecular motors play a key role in supporting the efficient delivery of myelin and other intracellular materials/organelles for proper axonal myelination.

### Myosin Motors in Myelination

Although an increasing number of studies have recognized a role for actin cytoskeleton in the formation of processes by Schwann cells and oligodendrocytes, and efficient axon wrapping during myelination [57–60], the role of myosin motors in myelination remains poorly understood. Wang and colleagues provided the first evidence that myosin II, which localizes to axons and synaptic terminals [61], participates in axon myelination in both CNS and PNS, although with opposite effects [13]. Treatment of Schwann cells in co-culture with dorsal-root ganglia (DRG) neurons (a representative in vitro model of the PNS) with the myosin II inhibitor blebbistatin decreased the number and size of segments positive for MBP, and the expression of myelin-related proteins (MBP, myelin protein zero, and MAG), indicating reduced myelination by Schwann cells. These defects were consequent to a perturbation in the protrusive actin activity required to establish the polarity of Schwann cells and to guide proper axon contact. By contrast, knockdown or inhibition of myosin II increased the number and size of segments positive for MBP in DRG neurons co-cultured with oligodendrocytes [13]. Taken together, these data indicate that myosin II activity, by regulating the actin network, is required for myelination: in Schwann cells, it works as an activator, whereas in oligodendrocytes, it acts as a negative regulator.

Despite this evidence, whether the active transport of neuronal cargo by myosin motors could regulate myelination remained uncharacterized. Based on immunoprecipitation analysis from mouse brain, Calliari and colleagues [62] reported an association between myosin Va and the RNA-binding protein HuD. Since HuD protein and myosin Va colocalize in cultured neurons and myelinated fibers of medullary roots, it strongly suggests that the neuronal transport of RNAs by myosin motors, which is required for proper cell polarization [63, 64], might be required for axon myelination. Nevertheless, it remains an open, attractive question for further studies.

### Kinesin Motors in Myelination

In oligodendrocytes from the *taiep* rat model (myelin mutant that shows a failure in CNS myelination, resulting in a progressive neurological disorder) [65], the aberrant accumulation of microtubules in their processes resulted in a perturbation of RNA trafficking [66]. These defects were partially suppressed following nocodazole treatment (to disrupt the microtubule network) [66], suggesting that an aberrant function of motor proteins that walk along microtubules could contribute to the RNA trafficking phenotype seen in the *taiep* rat model. This is in line with observations made later in the 1990s, which implicated kinesins in the transport of RNA granules along oligodendrocyte processes, as microtubule destabilization with nocodazole or suppression of kinesin expression with antisense oligonucleotides inhibited RNA trafficking [67]. Other studies analyzing mRNA purified from the myelin fraction of homogenized rat brain have shown a particular enrichment for the mRNA of three kinesins, KHC, KIF2, and KIF1A [68, 69]. These data suggest that kinesin motors could be key regulators of myelination.

In support of this hypothesis, several studies provided in vitro and in vivo evidence on the involvement of kinesins in myelination [10, 11, 70–74]. In Schwann cells, KIF13B was shown to interact and transport Discs large 1 (Dlg1; also known as synapse-associated protein 97 or SAP97, is a member of a family of proteins collectively named membrane-associated guanylate kinase homologs that, in humans, is encoded by the SAP97 gene) at sites of membrane remodeling. This is essential to support myelination, as KIF13B-delivered Dlg1 allows Sec8 (a component of the exocyst complex that has been implicated in tethering of secretory vesicles to specific regions on the plasma membrane)-dependent differentiation of myelinating cells and membrane formation. Consistent with this mechanistic theory, knock-down of KIF13B, Dlg1, or Sec8 dramatically reduced active myelination [11]. This indicates that kinesin KIF13B transports Dlg1 to myelination sites, where it interacts with Sec8 to support active myelination by Schwann cells. This hypothesis was further supported by another study, where the silencing of Dlg1 in Schwann cells resulted in thicker myelin segments, supporting a role for Dlg1 as a negative regulator of myelination [72]. More recent studies indicated that the negative regulation of myelination by Dlg1 involves the activation of the phosphatidylinositol 3-kinase (PI3K)/v-AKT murine thymoma viral oncogene homolog (AKT) pathway [74]. However, Dlg1 has opposite effects in the PNS and CNS. Whereas in the PNS Dlg-1-dependent activation of AKT pathway limits myelination, in the CNS, the activation of this pathway by Dlg1 favors active myelination [74]. Overall, these data suggest an active role for KIF13B, a member of the kinesin-3 superfamily in the transport of Dlg1 to support the homeostatic regulation of myelination.

Accordingly, severe CNS hypomyelination was also observed in KIF14 knockout mice [73], implicating this member of the kinesin-3 superfamily in myelination. However, the mechanisms involved in the regulation of myelination by KIF14 remain unexplored.

In zebrafish, a loss-of-function mutation in *kif1b*, the *kif1b*^*st43*^, was linked to perturbed transport of *mbp* mRNA in the CNS and defective outgrowth of the posterior lateral line nerve in PNS [70]. *kif1b*^*st43*^ mutant animals also showed a reduction in the number of PNS neurons. KIF1b activity was also shown to be required for localizing *mbp* mRNAs and other mRNAs to oligodendrocyte processes to produce the required amount of myelin around the axons, and to prevent ectopic generation of myelin-like membrane [10]. Using the same *kif1b*^*st43*^ mutant allele, Almeida and Lyons [71] also reported a dramatic reduction in the growth of reticulospinal axons in the posterior spinal cord. This created a region devoid of the initial targets for axon myelination, where a reduced number of oligodendrocytes was detected, consequent to a reduced proliferation and survival of oligodendrocyte progenitor cells (OPC) [71]. This indicates that KIF1B activity is required to adequately support axon growth and that signals coming from myelinated axons are essential to support proper OPC survival and proliferation. Overall, these data suggest that kinesin motors are active regulators of myelination, both in vivo and in vitro.

In line with this hypothesis, other studies have shown that the role of mTOR in the control of myelination involves KIF1B activity [59]. Acute inhibition of mTOR with rapamycin in OPCs significantly reduced KIF1B expression. Similarly, reduced expression of *kif1b* RNA and MBP protein was reported in O4-positive oligodendrocytes acutely isolated from spinal cords of mTOR knockout mice. These defects compromised the delivery of MBP to places where it is required for homeostatic myelination [59]. Overall, these data suggest that the reduction in myelin sheath tickness and quantity seen following mTOR inhibition [75, 76] may involve compromised KIF13B activity.

### Dynein Motor in Myelination

In line with the observations made for kinesin motors, multiple roles for dynein have been increasingly recognized in myelination.

Analysis of messenger ribonucleic acid (mRNA) purified from the myelin fraction of homogenized rat brain has shown a particular enrichment for dynein light intermediate chain (DLIC) 2 mRNA [68, 69]. Following this observation, early in the century, Song and colleagues [66] showed an inhibition of RNA trafficking in oligodendrocytes by reducing microtubule density with nocodazole or inhibiting dynein activity with anti-dynein antibodies. These studies provided strong evidence that the retrograde motor dynein may participate in myelination by transporting myelin components to sites of myelin sheath assembly [66].

Research in zebrafish has shown that dynein plays a role in the myelination of axons [12, 55, 77]. The Dynein Subunit Dynein Heavy Chain 1 (Dync1h1) mutant *dync1h1*^*hi3684Tg*^ shows defective myelination of peripheral nervous system (PNS) axons [12]. Specifically, mutant animals showed no expression of myelin basic protein (Mbp) posterior lateral line nerve (motor axons). Since Schwann cell progenitors reached motor roots in mutant larvae, this suggests a local requirement for dynein in Schwann cell-mediated myelination. Accordingly, in *dync1h1* mutant animals transplanted at blastula stage with *wild-type* cells, mutant Schwann cells supported normal axon wrapping, and axonal expression and localization of Mbp [12]. This indicated that dynein function is required locally in the axon to support PNS myelination in zebrafish. Similar observations were later made in zebrafish oligodendrocytes [55]. Specifically, *dync1h1* knockdown prevented the proliferation of the OPC population through cell division and diminished the stability of myelin sheaths. Additionally, a loss-of-function mutation in the *dync1h1* gene was linked to abnormal oligodendrocyte development [55]. This study in zebrafish demonstrated that Dync1h1 is crucial for regulating oligodendrocyte number and ensuring stable axon ensheathment by the oligodendrocyte membrane.

More recently, the dynein/dynactin motor complex was clearly implicated in the transport of *mbp* mRNAs in oligodendrocytes [77]. A forward genetic screen associated mutations in *actr10* (encodes the Arp11 subunit of dynactin), to a reduced density of myelinated axons and *mbp* mRNA localization in oligodendrocyte processes of zebrafish CNS. As *mbp* mRNA granules established a high-affinity association with the dynactin subunit p150^Glued^, dynein intermediate chain (DIC), and Arp11, it can be hypothesized that the impaired myelination phenotype observed in *actr10* mutant animals may be related to defective dynein/dynactin function. In support of this hypothesis, acute dynein inhibition with ciliobrevin D greatly impaired the *mbp* RNA transport in oligodendrocyte processes [77]. This is in line with other observations in mammalian neurons, where microRNA (miRNA)-mediated p150^Glued^ knockdown, which compromises dynactin/dynein function, impaired the axonal trafficking of node proteins [78]. Overall, these data indicate that dynein function is widely required for the transport of myelin components along the processes of myelinating cells, and locally necessary in axons to support myelination.

Supporting a role for dynein motor complex in myelination, previous studies have shown a predominant expression of the dynein cofactor, Nuclear Distribution E Homolog 1 (NDE1) in proliferating neuronal progenitors and migrating neurons during mouse embryogenesis [79, 80]. In line with these observations, Shimizu and colleagues [81] reported, in vitro, compromised process formation by oligodendrocyte and neuronal myelination following NDE1 depletion. This phenotype was rescued by exogenous expression of wild-type NDE1, but not by a mutant NDE1 unable to interact with the DIC component of the dynein complex [81]. This indicates that NDE1 is involved in oligodendrocyte morphological differentiation and that its role is dependent on the NDE1-DIC interaction.

In another study, Myllykoski and colleagues [82] identified dynein light chain DYNLL1 (also called dynein light chain 8) as a binding partner of L-myelin-associated glycoprotein (MAG) in a yeast two-hybrid screen. This interaction was specific for L-MAG, as no binding between S-MAG and DYNLL1 was detected in the same experimental setup [82, 83]. Further experiments with recombinant proteins have established an high-affinity association between these partners [82]. Overall, these data indicate that dynein function is required to support the proper development of myelinating cells and local axon wrapping, and for the trafficking of myelin sheath components to myelination sites.

## Demyelinating Diseases: a Brief Overview

Axon myelination in CNS and PNS neurons is of utmost importance to facilitate the propagation of electrical signals and to support axonal metabolism [7]. As an essential axonal component, conditions that result in damage to the myelin sheath that surrounds nerve fibers result in a wide spectrum of conditions, collectively named demyelinating diseases [17].

CNS demyelinating diseases are primarily characterized by demyelination of CNS neurons and, according to their pathogenesis, they can be classified into different categories: demyelination due to inflammatory processes (MS, neuromyelitis optica, transverse myelitis, acute disseminated encephalomyelitis, and acute hemorrhagic leukoencephalitis), viral demyelination (progressive multifocal leukoencephalopathy), demyelination caused by acquired metabolic derangements (central pontine and extrapontine myelinolysis and adrenoleukodystrophy/adrenomyeloneuropathy), hypoxic-ischemic forms of demyelination, and demyelination caused by focal compression [14–16].

By contrast, PNS demyelinating diseases arise from inappropriated myelination of PNS neurons, which translates into a wide spectrum of neuropathologies. Primary, inherited peripheral demyelinating neuropathies include Charcot-Marie-Tooth disease type 1 (CMT1), Dejerine-Sottas syndrome, congenital hypomyelinating neuropathy, and hereditary neuropathy with liability to pressure palsy. Alternatively, acquired demyelinating diseases of PNS include Guillain–Barre syndrome, anti-MAG neuropathy, chronic inflammatory demyelinating polyradiculoneuropathy, and POEMS syndrome (acronym of its multiorgan features: Polyneuropathy, Organomegaly, Endocrinopathy, M protein, and Skin changes) [15, 18].

Such conditions involve substantial damage to neuronal axons and myelinating cells, resulting in axonal degeneration and neuronal as likely consequences. Thus, these pathologies lead to a large spectrum of clinical manifestations as a consequence of defective communication between neurons and effector cells, including alterations in locomotion or sensorial perception [17].

## Molecular Motors in the Pathogenesis of Neurodegenerative Diseases

Neurodegenerative diseases encompass a large group of diseases characterized by progressive degeneration of neurons in the CNS or PNS. These conditions, including Alzheimer’s, Parkinson’s, and Huntington’s diseases, and amyotrophic lateral sclerosis, among others, result in debilitating symptoms that impair motor function, cognition, and/or behavior. It has been increasingly recognized that dysfunctional trafficking of intracellular organelles and other cellular materials is involved in the pathogenesis of several neurodegenerative disorders [5, 84–86], including demyelinating diseases and other neuropathies [19].

In Alzheimer’s and Parkinson’s diseases, the two most prevalent neurodegenerative diseases worldwide, the involvement of dysfunctional intracellular transport machinery in the pathophysiology of these conditions has been clearly revealed [87, 88]. Studies have shown dysfunctional mitochondrial distribution in neurons from Alzheimer’s disease patients [89, 90] and in transgenic Tg2576 mice, which express the amyloid precursor protein (APP) [91]. Additionally, abnormal phosphorylation of tau detected in the brains of Alzheimer’s disease patients result in microtubule structural changes [92], and amyloid β peptides, a hallmark of the disease, influence the post-translational modifications of microtubule proteins, disrupting the balance between microtubule assembly and disassembly [93, 94]. In Parkinson’s disease, axonal transport defects have been observed even in the early stages of the disease, which include altered levels of kinesin and dynein [95], and tau hyperphosphorylation [96].

Overall, it becomes clear that axonal transport defects, likely resulting from altered function of motor proteins, are implicated in the pathogenesis of neurodegenerative diseases. These defects can arise from alterations in dynein, kinesins, or myosins, their adaptors, or their walking tracks, often causing erroneous binding of motor proteins to cargo or impaired function, instability of the microtubule/actin cytoskeleton, or inaccurate recruitment of cargo adaptors. Mutations in genes that code for components of molecular motors also suggest that disruptions in intracellular transport can accelerate neurodegeneration [97–102].

### Molecular Motors in Multiple Sclerosis

MS represents a chronic autoimmune, inflammatory neurological disease and the most common and investigated CNS demyelinating disease, constituting the leading cause of neurological disability in young adults. It affects about 2.1 million people worldwide, greatly impacting patients’ quality of life and representing strong economic and health concerns for society [103]. Although its etiology remains poorly characterized, it is believed to result from a combinatorial effect of genetic factors and environmental stressors. The clinical manifestations of the disease are variable among individuals and include muscular weakness, fatigue, paresthesia, optic neuritis, trigeminal neuralgia, abnormal autonomic motor control of bladder and bowel, and psychologic/psychiatric manifestations. The most characteristic pathological hallmark of the disease is the focal demyelination, clinically known as plaques, which show a characteristic inflammation and gliosis [104]. These lesions can be dispersed throughout the entire CNS, but particularly frequent in the brainstem, spinal cord, cerebellum, and optic nerve. Those lesions are particularly evident by magnetic resonance imaging, where the axonal degeneration consequent to myelin damage constitutes the main cause for the progression of the irreversible neuronal destruction that leads to permanent disability. Typically, the onset of the disease is characterized by the gradual recovery of symptoms, the relapsing remittent stage (RRMS), which naturally progresses 10–15 years after RRMS onset to secondary progressive MS (SPMS), where discrete relapses account for disease progression. The most severe form of the disease is the primary progressive MS (PPMS), which affects approximately 10–15% of MS patients and is characterized by a progressive worsening of the disease from its onset [17, 105, 106].

Impaired axonal transport represents one of the first pathological signs observed in the experimental autoimmune encephalomyelitis (EAE) animal model of MS, even before the instauration of any structural abnormalities of axons, cargos, or microtubules. In a chronic EAE model, the transport of mitochondria in both anterograde and retrograde directions was significantly diminished even prior to the onset of demyelination, and this reduction persisted for several weeks [107]. Furthermore, in vitro studies also indicated abnormal transport of mitochondria in demyelinated axons [108]. This is consistent with the observations indicating axonal transport of mitochondrial at a uniform speed in myelin-deficient rats [109]. In addition, mice with a null mutation in the *Plp* gene, which codes for proteolipid protein 1, a predominant component of myelin, showed compromised anterograde and retrograde transport within axons [110] and microtubule abnormalities [111]. Another key indicator of defective axonal transport is the deposition of visible aggregates of organelles and proteins within the axon, leading to axon swelling. In healthy neurons, the APP is efficiently transported along the axon to the synapse [112]. However, *postmortem* analyses of human MS brains have revealed axonal APP accumulation, indicating an impairment in the axonal transport machinery [113]. Notably, APP deposits could be detected in early stages of the disease, and the number of APP-positive axons increased with disease duration [114].

Neuroinflammation represents another key aspect in MS that may influence the intracellular transport machinery in the context of this disease [104]. The pro-inflammatory cytokine tumor necrosis factor α (TNF-α), which is notably increased in the CNS of MS patients, correlated directly with the severity of the disease [115], has been shown to alter microtubule stability [116–118], thereby influencing the intracellular transport dynamics. Currently, although the precise mechanism by which TNF-α causes microtubule destabilization is not fully understood, this cytokine is known to promote the phosphorylation of c-Jun N-terminal kinase, which leads to the dissociation of KIF5B from microtubules [119].

Another hypothesis suggests that the effects of TNF-α on microtubules might involve glutamate toxicity, as TNF-α stimulates glutamate release and reduces its uptake by glial cells [120]. Under normal conditions, glutamate binds to channels on the cell membrane, causing controlled ion flow. Overstimulation of these channels by glutamate leads to increased ion flux, elevating intracellular Ca^2+^ levels, which activates intracellular cascades, culminating in apoptosis [121]. Even before reaching apoptotic levels, elevated Ca^2+^ levels disrupt the cytoskeleton by destabilizing microtubules [122] and intermediate filaments [123]. In fact, the axonal degeneration characteristic of MS has been associated with intracellular Ca^2+^ overload [124], and in in vitro experiments with neurons in culture, glutamate-induced Ca^2+^ influx impaired fast axonal transport [125]. Particularly for mitochondria, it is well established that an increase in Ca^2+^ levels negatively impacts their anterograde transport, as Ca^2+^ binding to the Rho-like GTPase elongation factor (EF) domains of the mitochondrial protein Miro induces a conformational change to an inefficient state [126, 127]. Therefore, it can be speculated that glutamate may underlie the increased Ca^2+^ influx observed in demyelinated axons, thereby influencing axonal transport. In addition, the glutamate-dependent effects of elevated Ca^2+^ levels in destabilizing intermediate filaments help to explain why around half of demyelinated axons in the brain of MS patients exhibited neurofilament network fragmentation [128]. All these events support the increasingly recognized involvement of glutamate excitotoxicity in the pathogenesis of MS [129–132].

In addition to the effects of glutamate on microtubule stability, it can also alter the pattern of posttranslational modifications (PTMs). Along with the impact on microtubule dynamics, PTMs can also alter the binding affinity of molecules to microtubules, including motor proteins, thereby influencing microtubule-based transport [133–136]. For instance, a combination of PTMs such as acetylation and detyrosination enhances Kinesin-1 (Kif5) binding to microtubules, its motor activity, and its preferential targeting to the axon [137–139]. However, it remains unclear whether alterations in PTMs due to glutamate excitotoxicity are significant in the context of MS.

In neuroinflammation, activated microglia not only produce TNF-α and other cytokines, but also express the enzyme inducible nitric oxide synthase (iNOS). Nitric oxide (NO), produced by iNOS, is recognized for disrupting the microtubule network by nitrosylating MAP1B. This process leads to growth cone collapse and axon retraction [140]. This indicates that several alterations observed under neuroinflammatory conditions may influence microtubule-dependent axonal transport. However, despite this substantial body of evidence linking defects in the transport machinery to MS, some of the findings are indirect. Based on this, below we provide a comprehensive overview of studies directly implicating alterations in molecular motors in the pathogenesis of the disease.

### Cell Biology and Genetic Studies Directly Associating Molecular Motors with Multiple Sclerosis

**Fig. 3:**
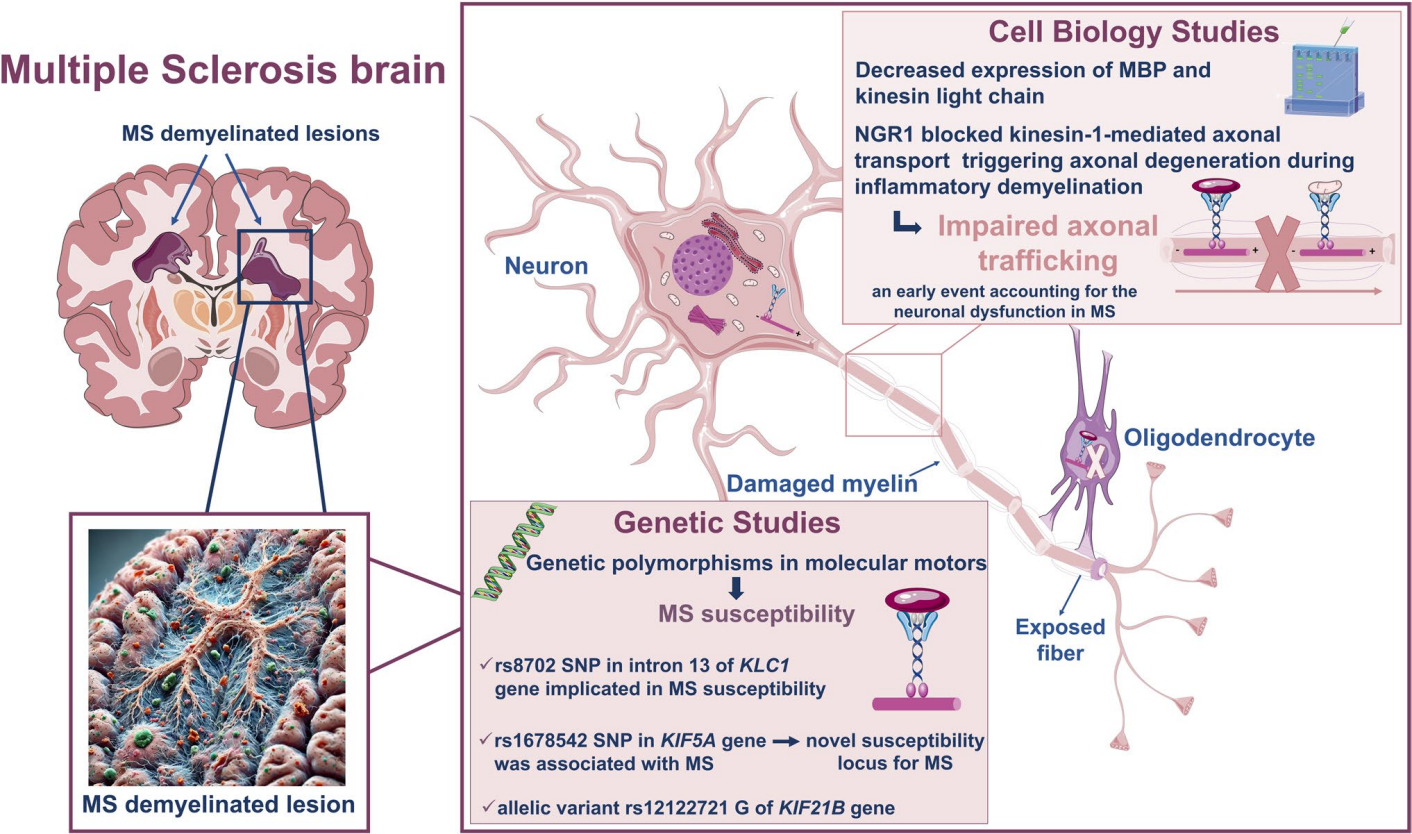
Dysfunction of the molecular motors’ machinery in multiple sclerosis. The involvement of molecular motors, particularly kinesins, in the etiology and pathogenesis of multiple sclerosis (MS) has been suggested by cell biology and genetic studies. Cell biology studies have revealed decreased levels of myelin basic protein (MBP) and Kinesin Light Chain in animal models of MS. Genetic studies in MS patients have established an association between genetic polymorphisms in components of molecular motors and MS. Such predisposing polymorphisms include the rs8702 single nucleotide polymorphism (SNP) in intron 13 of kinesin light chain 1-coding gene (*KLC1*), the rs1678542 SNP in *KIF5A* gene, and the allelic variant rs12122721 G of the *KIF21B* gene. The association between the rs10492972 polymorphism in the *KIF1B* gene and MS susceptibility remains controversial

The involvement of molecular motors, particularly kinesins, in the etiology and pathogenesis of MS has been suggested by cell biology studies in animal models and genetics studies in MS patients. The demyelination seen in the hippocampus of the cuprizone animal model of MS is accompanied by decreased expression of MBP and kinesin light chain, but not changes in dynein expression [141]. Nevertheless, because hippocampal demyelination is not accompanied by obvious neuronal loss [141], it suggests that an impairment in the axonal trafficking machinery could represent an early event accounting for the neuronal dysfunction in MS. Such hypothesis was further supported by Lee and colleagues [142], showing that Nogo-66 receptor (NGR1), by controlling kinesin-1 vesicular transport, works as a negative regulator of axonal degeneration in the context of neuroinflammation. In the experimental autoimmune encephalomyelitis (EAE) model, neuronal depletion of NGR1 resulted in a significant delay in the onset of EAE. Detailed mechanistic analysis revealed that NGR1 triggers the dissociation of kinesin-1 from the vesicular cargo protein, collapsing response mediator protein 2 (CRMP2), thus resulting in overt demyelination. Axonal depletion of NGR1, by preserving the kinesin-1-CRMP2 association, limited oligodendrocyte dystrophy and preserved myelin integrity during EAE. Overall, these data indicated that NGR1 triggers axonal degeneration during inflammatory demyelination by blocking kinesin-1-mediated axonal transport [142]. In addition, this directly implicates the spatial distribution of cargo by motor proteins, particularly kinesin-1, in axon demyelination under neuroinflammatory conditions, such as those observed in MS. The main findings from cell biology studies linking dysfunctional molecular motor machinery to MS are illustrated in Fig. 3.

Over the years, several genetic studies have also explored whether allelic variants of genes coding for molecular motor proteins could be associated with a higher predisposition for developing MS (Fig. 3; Table 2).

**Table 2:** Components of molecular motors affected by genetic mutations in multiple sclerosis

Molecular motor	Mutated gene	Common protein name	Allelic variant	Samples	Main results	Reference
Kinesin	*KLC1*	Kinesin Light-Chain 1	rs8702	102 patients with relapsing remitting MS and 207 controls	rs8702 CC carriers showed a lower susceptibility for MS (*p* < 0.05)	[143]
Kinesin	*KIF1B*	Kinesin-like protein 1B	rs10492972 C	2679 MS patients (535 Dutch, 826 Swedish, and 1318 Canadians) and 3125 healthy controls (621 Dutch, 997 Swedish, and 1507 Canadians)	rs10492972 C carriers showed a higher risk of developing MS (*p* = 2.5 × 10^−10^)	[144]
Kinesin	*KIF1B*	Kinesin-like protein 1B	rs10492972 C	2137 trio families (both parents and a child with the disease), 8391 MS cases, and 8052 unrelated controls from Australia, Belgium, Finland, Italy, Norway, Sweden, Great Britain, and the USA	No association between the allelic variant rs10492972 C and MS susceptibility	[145]
Kinesin	*KIF1B*	Kinesin-like protein 1B	rs10492972 C	222 patients with PPMS or PRMS and 221 healthy controls of Italian origin	rs10492972 C carriers did not show neither a higher risk of developing PPMS or PRMS nor significant differences in the rate of disability progression	[146]
Kinesin	*KIF1B*	Kinesin-like protein 1B	rs10492972	833 MS patients and 689 healthy controls from Russia	No significant association for the frequency of the genotypes TT, CC, or TC of the polymorphism rs10492972 in the *KIF1B* gene between control and RRMS, PPMS and SPMS subpopulations of MS patients No association between this polymorphism and MS characteristics (age of onset, duration, or progression of the disease)	[147]
Kinesin	*KIF1B*	Kinesin-like protein 1B	rs10492972	3874 MS patients and 5723 healthy controls from Australia and New Zealand	Non-significant association between the rs10492972 polymorphism and MS susceptibility (*p* = 1.0)	[148]
Kinesin	*KIF1B*	Kinesin-like protein 1B	rs10492972 C	609 MS patients and 230 controls from Greek origin	No association of the rs10492972 C allelic variant with MS; among MS patients, this polymorphism showed no association with disease progression	[149]
Kinesin	*KIF21B*	Kinesin-like protein KIF21B	rs12122721 G	931 MS trio families (both parents plus a child with the disease), 3507 isolated cases of MS, and 8024 controls from the USA and the UK	rs12122721 G polymorphism was associated with a higher susceptibility to develop MS (*p* = 6.56 × 10^−10^)	[150]
Kinesin	*KIF21B*	Kinesin-like protein KIF21B	rs12122721 G	791 MS patients and 1098 unrelated controls from Belgium	rs12122721 G carriers showed a higher risk of developing MS (*p* = 0.01)	[151]
Kinesin	*KIF5A*	Kinesin-like protein KIF5A	rs1678542	2864 MS patients and 2930 controls from Spain	rs1678542 SNP in *KIF5A* gene was associated with MS (*p* = 0.001)	[152]

In 2007, Szolnoki and colleagues [143] provided the first evidence that genetic polymorphisms in molecular motors are associated with MS susceptibility. In a cohort of 102 RRMS patients and 207 neuroimaging alteration-free controls, the rs8702 single nucleotide polymorphism (SNP) in intron 13 of the kinesin light chain 1 (*KLC1*) gene was implicated in MS susceptibility, with CC carriers being significantly protected from developing the disease (*p* < 0.05) [143]. This study provided, therefore, strong evidence for a link between polymorphisms in genes coding for components of molecular motors and MS susceptibility.

Supporting this hypothesis, in a cohort of 2679 MS patients (535 from the Netherlands, 826 from Sweden, and 1318 from Canada) and 3125 healthy controls (621 from the Netherlands, 997 from Sweden, and 1507 from Canada), Aulchenko and colleagues [144] further showed that the allelic variant rs10492972 C, located in the *KIF1B* gene, was associated with a higher risk for developing MS (*p* = 2.5 × 10^−10^) [144]. However, other studies using large cohorts of MS patients from various countries around the world, including trio families (both parents and a child with the disease) [145], as well as patients with PPMS, SPMS, or PRMS [146, 147], found no association between this allelic variant and MS susceptibility [145–149], nor with disease characteristics such as age of onset, disease duration, or progression [146, 147]. Based on these studies, it is difficult to establish an association between the rs10492972 polymorphism in the *KIF1B* gene and MS susceptibility or disease characteristics.

On the other hand, genetic studies on polymorphisms in genes coding for *KIF5A* and KIF21B have provided a more clear and consistent association with MS [150–152]. In a cohort of 2864 MS patients and 2930 controls from Spain, the rs1678542 SNP in *KIF5A* gene was associated with MS (*p* = 0.001) [152]. In a study analyzing more than 30 000 SNPs in 931 MS trio families (both parents plus one child with the disease), 3507 isolated MS cases, and 8024 controls from the USA and the UK, the allelic variant rs12122721 G of *KIF21B* gene was reported as a novel susceptibility locus for MS (*p* = 6.56 × 10^−10^) [150]. Similarly, in a Belgian study population of 791 MS patients and 1098 unrelated controls, the same allelic variant, rs12122721 G, of the *KIF21B* gene was also associated with a higher risk of MS (*p* = 0.01) [151].

Thus, although the actual data make a clear association between polymorphisms in *KLC1*, *KIF5A*, and *KIF21B* genes and MS, the same remains inconclusive for allelic variants of the *KIF1B* gene. A deeper understanding of the genetic determinants of MS would help in establishing a clear role for genetics in the etiology of the disease and in identifying subpopulations with higher susceptibility for developing the disease. In addition, with the advent of genetic engineering, such knowledge could leverage the development of novel therapies, including genome editing, particularly oriented toward undiagnosed individuals at high risk.

## Conclusion

The intracellular transport of organelles and materials is powered by molecular motors and is coupled to a variety of functions in dividing and non-dividing cells. Kinesins predominantly transport cargo toward microtubule plus-ends, influencing microtubule dynamics and organelle positioning, while dynein facilitates minus-end directed transport, essential for organelle distribution and cell division. On the other hand, motor and non-motor myosins are pivotal in muscle contraction and are responsible for orchestrating changes in the actin cytoskeleton to guarantee cellular movement, membrane trafficking, and signaling. Neuronal cells, due to their structure and specialized function, are highly dependent on the intracellular transport machinery for the delivery of components synthesized in the soma along their long processes to their final destinations. This mechanism is particularly relevant for human health, as dysfunctional control of intracellular transport of cargoes has been linked to a large spectrum of brain pathologies, including neurodegenerative and neurological diseases, highlighting a pivotal role of intracellular transport in brain homeostasis.

The involvement of molecular motors in axon myelination has been recognized since the very end of the last millennium. Dynein motor is prominently involved in retrograde transport within oligodendrocytes and Schwann cells, crucial for positioning the myelin components and regulating myelination. This process is exemplified by studies in zebrafish and mammalian models, where dynein dysfunction leads to severe myelination defects, highlighting its essential role. Similarly, kinesins contribute significantly to myelination dynamics, with studies implicating various kinesin types in RNA transport and the delivery of myelin protein, essential for proper myelin formation and maintenance. The involvement of myosins in myelination remains less explored but emerging evidence suggests potential roles for them in Schwann cell and oligodendrocyte function, influencing axon ensheathment and myelin formation. However, these mechanisms remain poorly characterized, anticipating that molecular motors could have multiple other roles during axon myelination.

Supporting a role for molecular motors in myelination, dysregulation of these molecules is implicated in MS and other demyelinating diseases. Disrupted kinesin-mediated axonal transport is linked to MS pathogenesis, contributing to the neuronal dysfunction and progressive disability characteristic of the disease. Similarly, aberrant dynein function in oligodendrocytes and Schwann cells has been associated with impaired myelin maintenance and axonal support in both experimental models and clinical studies. In complement, genetic studies have linked polymorphisms in genes coding for components of molecular motors to a higher risk of developing MS.

Understanding the roles of molecular motors in myelination not only helps in the elucidation of fundamental cellular processes but also has potential for identifying novel therapeutic strategies. Targeting these motors and their regulatory pathways could offer novel strategies for restoring proper axonal function and mitigate the progression of the disease.

## Future Perspectives

### How Molecular Motors Function and Are Regulated

As our understanding of molecular motors continues to progress, future research will likely uncover deeper insights into their mechanisms and regulatory pathways. Despite significant efforts in characterizing motor proteins like kinesins, dynein, and myosins, many aspects of their function remain uncharacterized. For instance, understanding the precise mechanisms by which motor complexes interact with their cargoes and mediate transport through the cytoplasm, which is full of components, remains a fundamental challenge. Advanced imaging techniques, such as super-resolution microscopy [153, 154] and cryo-electron microscopy [155–158], have made it possible to visualize these processes at unprecedented resolution. In addition, the implementation of expansion microscopy methodologies [159, 160], which physically expands biological specimens to achieve nanoscale resolution while preserving spatial relationships between molecules, may provide important insights into the dynamics of motor-cargo interactions and elucidate the molecular determinants that govern specificity and directionality in motor-driven transport.

### Role of Molecular Motors in Demyelinating Diseases

The role of molecular motors in neurodegenerative diseases, particularly demyelinating disorders like MS, rises interesting unanswered questions for future investigation. Emerging evidence suggests that dysregulation of motor proteins contributes to the pathophysiology of these diseases [19]. It has become clear that mutations in kinesins and dynein are implicated in compromised intracellular trafficking and organelle transport, processes critical for maintaining neuronal homeostasis. However, in demyelinating diseases, it remains difficult to draw a clear-cut conclusion about whether dysfunctional activity of molecular motors is the primary cause or just a consequence of demyelination. Some strategies that could be useful to answer this question include modifying the activity of molecular motors either by genetic (e.g., knockdown or knockout experiments using RNA interference or CRISPR-Cas9) or pharmacological methods (using specific inhibitors or activators) and analyzing the degree of demyelination using commonly implemented techniques, such as electron microscopy or magnetic resonance imaging, in animal models. A major concern is the requirement of molecular motors during developmental stages, which could complicate the analysis in more complex and developed models.

Another intriguing question that remains uncharacterized is how intracellular transport is reprogrammed to help neurons cope with demyelination and during remyelinating stages. At this level, transcriptomic screenings may provide important cues about changes in gene profiles related to transport machinery during these processes. Additionally, proteomics, particularly phosphoproteomics, can analyze the phosphorylation states of motor proteins and associated regulators during myelination, demyelination, and remyelination, thereby helping to understand potential post-translational modifications that affect transport.

### The Putative Interplay Between Molecular Motors and Immune Cells in Demyelinating Diseases

Neuroinflammation makes a substantial contribution to the pathophysiology of some demyelinating diseases, particularly relevant to MS [104]. However, the interplay between molecular motors and immune cells, as well as the connection between neuroinflammation and motor protein dysfunction, remains poorly characterized. Developing co-culture systems where neurons are cultured with immune cells, such as microglia or astrocytes, may allow for the study of potential direct interactions between these cells and how the function of molecular motors is affected under inflammatory conditions.

Elucidating these questions may provide transformative solutions in the field of neurodegeneration. Strategies aimed at modulating the activity of motor protein or enhancing their transport efficiency could offer novel therapeutic interventions. Small molecules, gene therapies, or biologic molecules designed to selectively regulate motor function hold promise for mitigating disease progression and promoting neural repair. Moreover, advances in drug delivery technologies, such as nanocarriers and targeted delivery systems, may facilitate the precise and efficient delivery of therapies targeting motor proteins to affected tissues.

## Data Availability

Not applicable.
